# AMPA and NMDA Receptors in Hippocampus of Rats with Fluoride-Induced Cognitive Decline

**DOI:** 10.3390/ijms252111796

**Published:** 2024-11-02

**Authors:** Olga Vladimirovna Nadei, Natalia Ivanovna Agalakova

**Affiliations:** Sechenov Institute of Evolutionary Physiology and Biochemistry, Russian Academy of Sciences, 44 Thorez Avenue, Saint-Petersburg 194223, Russia; olganadej@gmail.com

**Keywords:** rat, hippocampus, AMPARs, NMDARs, gene expression, subcellular distribution

## Abstract

This experimental study was performed to evaluate the alterations in the expression of a few subunits composing glutamate AMPA (a-amino-3-hydroxy-5-methyl-4-isoxazolepropionic acid) and NMDA (N-methyl-D-aspartate) receptors in the hippocampal cells of Wistar rats in response to long-term fluoride (F^-^) exposure. The animals were given water with background 0.4 (control), 5, 20, and 50 ppm F^-^ (as NaF) for 12 months. The cognitive capacities of rats were examined by novel object recognition (NOR), Y-maze test, and Morris water maze tests. RT-qPCR and Western blotting techniques were used to evaluate the expression of different AMPA and NMDA subunits at transcriptional and translational levels, respectively. Long-term F^-^ poisoning disturbed the formation of hippocampus-dependent working spatial and long-term non-spatial memory. The expression of *Gria1*, *Gria2*, and *Gria3* genes encoding different subunits of AMPA receptors were comparable in hippocampi of control and F^-^-exposed animals, although the levels of both *Grin2a* and *Grin2b* mRNA increased. Long-term F^-^ intake enhanced the ratio of phospho-GluA1/total-GluA1 proteins in subcellular fraction enriched with cytosolic proteins, while decreased content of GluA2 but elevated level of GluA3 were observed in subcellular fraction enriched with membrane proteins. Such changes were accompanied by increased phosphorylation of GluN2A and GluN2B subunits, higher ratios of GluN2A/GluN1 and GluN2B/GluN1 proteins in the cytosol, and GluN2A/GluN2B ratio in membranes. These changes indicate the predominance of Ca^2+^-permeable AMPARs in membranes and a shift between different NMDARs subunits in hippocampal cells of F^-^-exposed rats, which is typical for neurodegeneration and can at least partially underly the observed disturbances in cognitive capacities of animals.

## 1. Introduction

Fluoride (F^-^) is ubiquitously distributed in nature due to release from natural (volcanic and geothermal activity, and mineral leaching) and anthropogenic (coal burning, metals, and fertilizers processing) sources [1,2]. In endemic areas, the levels of naturally occurring F^-^ exceeding the permissible limit of 1.5 mg/L [3] are found in water available for daily human needs and farm animal livestock. In developed countries, F^-^-supplementation of drinking water, salt, milk, and dental products is the second important pathway of F^-^ intake. Chronic exposure to F^-^ at industrial sites or accidental poisoning with F^-^-containing chemicals at home are also not rare [1,2]. However, excessive F^-^ consumption induces a spectrum of health disorders, such as dental and skeletal fluorosis and damage to various soft tissues [1,3]. Most important, many recent works raised a concern on possible F^-^ neurotoxicity, including increased risk of neurodegenerative problems such as Parkinson’s disease and Alzheimer’s disease in adults [4,5,6,7], autism spectrum disorders, decreased Intelligence quotient (IQ) score and difficulties with attention in children [8,9,10,11], and susceptibility to brain tumors [12]. Other studies, however, claim an absence of thorough clinical investigations and convincing evidence in favor of the association between F^-^ intake and neurological disorders, while the existing findings are insufficient [13,14]. Such controversies highlight the necessity of new research focused on F^-^ neurotoxicity.

F^-^ is known to freely transit the hematoencephalic barrier and accumulate in brain structures to a degree directly reflecting the consumption rate (summarized in [5]). Our previous works [15,16] have shown that the development of chronic fluorosis in rats was accompanied by a decline in cognitive capacities, pathological alterations and death of neurons in CA1 and CA3 hippocampal fields, and altered expression of Ca^2+^-dependent signaling molecules implicated in long-term potentiation (LTP).

The processes of learning and memory formation are believed to be based on synaptic plasticity, whereas cognitive impairment is associated with perturbed excitatory neurotransmission. F^-^ poisoning alters the levels of glutamate and its receptors in the brains of rodents [17,18,19,20,21]. Glutamate receptors are clustered at postsynaptic membranes and classified by agonist selectivity as ionotropic AMPA (a-amino-3-hydroxy-5-methyl-4-isoxazolepropionic acid), NMDA (N-methyl-D-aspartate) or kainite receptors, and metabotropic glutamate receptors (mGluR) [22,23]. Tetrameric AMPARs are composed of various combinations of GluA1-GluA4 subunits encoded by *Gria1-Gria4* genes [24,25,26,27]. In excitatory neurons of the mature hippocampus, AMPARs predominantly exist as GluA1/2 and GluA2/3 heterodimers at approximately similar ratios. GluA2-containing AMPARs are impermeable to Ca^2+^, whereas that with GluA1 and GluA3 transit Ca^2+^ following activation. Being the central mediators of rapid excitatory glutamatergic synaptic transmission, GluA1-possessing AMPARs are inserted into synapses following LTP and play an important role in learning and memory. GluA2/3-containing AMPARs are not significantly involved in LTP or memory formation but modulates the synaptic strength.

NMDARs are implicated in long-term synaptic plasticity associated with transcription-dependent processes and global protein synthesis. Functional tetrameric NMDARs are highly permeable to Ca^2+^ and composed of two obligatory GluN1 subunits and two GluN2, GluN3 or GluN2/GluN3 subunits. GluN2A and GluN2B are the most abundant regulatory subunits in cognitive-related brain structures. GluN2A-containing NMDARs are located primarily at synaptic sites and are believed to implicate in protective pathways, whereas GluN2B-containing receptors are distributed mainly at extrasynaptic sites and increase neuronal vulnerability. Shifts in the GluN2A/GluN2B ratio underlie both learning and memory, but also different neuropathologies.

The goal of the present study was a thorough evaluation of possible changes in the expression of a few major subunits composing AMPARs and NMDARs in the hippocampus of rats with stable fluorosis at transcriptional and translational levels, as well as their subcellular distribution and phosphorylation state. The work was focused on the hippocampus because it provides the synaptic basis for the initiation, formation, and consolidation of memory.

## 2. Results

**Scheme 1:**
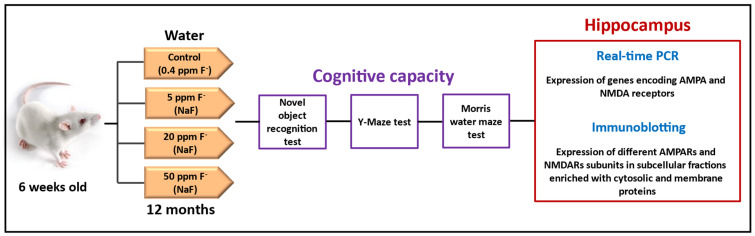
The timeline of entire experimental procedure.

The design of experiments is shown in Scheme 1.

### 2.1. Brain F^-^ Content

The average F^-^ content in the brains of animals that consumed regular drinking water was 0.43 ± 0.08 µg/g. In the brains of rats exposed to 5 and 20 ppm F^-^, its content increased to 1.23 ± 0.11 (*p* = 0.0019, n = 5) and 1.94 ± 0.18 µg/g (*p* < 0.0001, n = 5), respectively (F (2, 12) = 33.61, *p* < 0.0001) (mean ± SEM, one-way ANOVA, Bonferroni post-hoc test).

### 2.2. Influence of Excessive F^-^ Consumption on Memory and Spatial Learning

**Figure 1:**
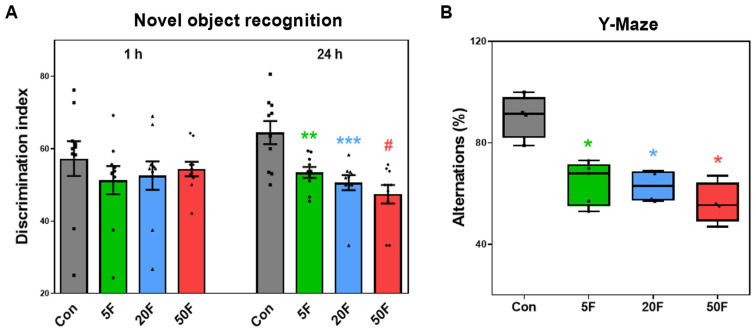
Effects of chronic F^-^ exposure on non-spatial memory. (**A**) Novel object recognition test. Average discrimination indexes calculated for rats from different groups in 1-h and 24-h trials. One-way ANOVA followed by Bonferroni multiple comparison test. Presented are mean values ± SEM (n = 10). (**B**) Y-maze test. Average values ± SEM (n = 5) of spontaneous alternations coefficient for animals from different groups. Mann-Whitney U-criterion. * *p* < 0.05, ** *p* < 0.01, *** *p* < 0.001, # *p* < 0.0001 compared to control. Con—control, 5F—5 ppm F^-^, 20F—20 ppm F^-^, 50F—50 ppm F^-^.

The non-spatial short-term and long-term memory of animals was evaluated by NOR (novel object recognition) test in 1 h and 24 h after training. The values of the discrimination index (DI) calculated for the 1-h session were comparable for rats from all groups, i.e., they clearly distinguished old and new objects (Figure 1A). In 24 h after training, however, DIs calculated for F^-^-poisoned rats were significantly lower than for control animals (*p* = 0.0080, *p* = 0.0008, and *p* < 0.0001 vs. control for 5F, 20F, and 50F group, respectively), indicating that they were not able to remember old object. These results resemble the data of our previous work [16], which also showed that excessive F^-^ consumption did not affect the formation of short-term memory but impaired long-term memory.

The working memory of rats was estimated by the Y-maze test. The values of spontaneous alternations coefficients calculated for rats given all three F^-^ doses were considerably lower than for control animals (*p* = 0.0159, *p* = 0.0286, and *p* = 0.0143 vs. control for 5F, 20F and 50F group, respectively) (Figure 1B). The total number of arms entries decreased statistically significantly in the groups of animals that consumed 20 and 50 ppm F^-^. These results indicate that acquisition of the spatial task and, accordingly, working memory was impaired by long-term F^-^ exposure.

**Figure 2:**
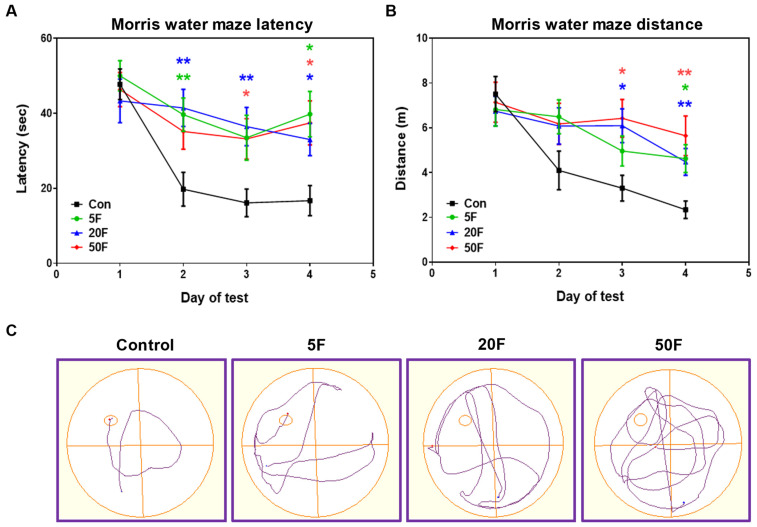
Hidden platform acquisition trial of MWM test. (**A**,**B**) Latency and distance traveled by rats to find the platform (means ± SEM) during four days of training. Two-way RM-ANOVA with Bonferroni post-hoc test. * *p* < 0.05, ** *p* < 0.01 in comparison to control (n = 10). (**C**) Representative trajectories obtained for one rat from each group on 4th day of the hidden platform test.

The spatial learning and formation of long-term memory were evaluated by the Morris water maze (MWM) test. On day 1 of the MWM hidden platform acquisition test, the rats from all groups, including the control, needed comparable time to locate the submerged platform (Figure 2A). A progressive decline in the latency of escape to the hidden platform was observed throughout the four-day training. However, two-way RM-ANOVA revealed a significant effect of group (F (3, 72) = 7.342; *p* = 0.0002) and effect of day (F (2.753, 161.5) = 11.42; *p* < 0.0001), but not significant group by day interaction (F (9, 176) = 1.568; *p* = 0.1281). Further comparison of averages calculated for each day has shown that F^-^-exposed rats needed considerably more time to find the escape platform than control animals. A similar tendency was observed for the distance traveled by rats to reach the platform (Figure 2B), although the statistically significant differences were revealed starting from day 3 only. On the last day of the hidden platform test, all rats from F^-^-treated groups exhibited a longer route before locating the platform, some animals were not able to find the platform at all within a 1-min trial (Figure 2C).

In the MWM spatial probe test (day 5 without platform), the rats from F^-^-treated groups needed more time to locate the target quadrant and traveled a short distance in this zone (Table 1). The speed of swimming was comparable in all groups of animals on each day of the experiment and on day 5 (Table 1). Thus, although the MWM test did not yield consistent and stable results, it showed that exposure of the rats to excessive F^-^ moderately impaired the processes of visuospatial learning and long-term memory formation.

### 2.3. Effect of Fluoride on Expression of Subunits Composing AMPA Receptors in Rat Hippocampus

**Figure 3:**
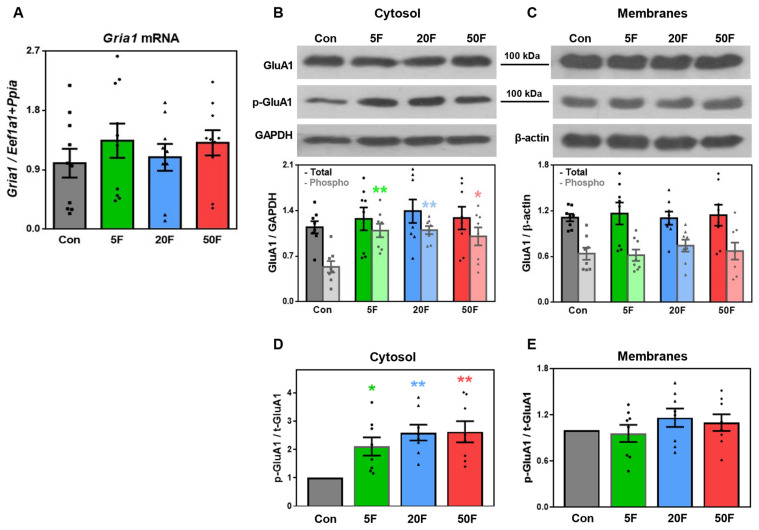
Expression of GluA1 subunits of AMPARs in hippocampus of F^-^ exposed rats at transcriptional and translational levels. (**A**) Relative expression of *Gria1* gene in hippocampus of rats consumed different F^-^ doses normalized to a pair of reference genes (*Eef1a+Ppia*) (average values ± SEM, n = 10). (**B**,**C**) Changes in the protein content of native and phosphorylated (Ser845) forms of GluA1 subunit in the cytosol and membranes of hippocampal cells, respectively. Shown are typical immunoblots and average values ± SEM (n = 8). * *p* < 0.05, ** *p* < 0.01 in comparison to corresponding (native or phosphorylated) control. (**D**,**E**) Changes in phospho-GluA1/total GluA1 ratios in cytosolic and membrane fractions. The data are expressed relative to p-GluA1/t-GluA1 ratio of respective controls, which is taken as 1 in each experiment (means ± SEM, n = 8). * *p* < 0.05, ** *p* < 0.01 in comparison to control ratios. One-way ANOVA followed by Bonferroni multiple comparison test.

The expression of the *Gria1* gene encoding GluA1 subunits of AMPA receptors was comparable in hippocampal tissues of control and F^-^-poisoned rats (Figure 3A). The content and subcellular distribution of the native form of GluA1 of AMPAR subunits did not change in the cellular fractions enriched with either cytosolic or membrane proteins (further referred to as cytosol or membranes, respectively) (Figure 3B,C). However, the expression of the phosphorylated (Ser845) form of GluA1 subunits increased in the cytosolic fraction of hippocampal cells of animals consumed all F^-^ doses (*p* = 0.0017, *p* = 0.0016 and *p* = 0.0089 vs. control for 5F, 20F, and 50F, respectively) (Figure 3B). Accordingly, the ratios of phospho- to total GluA1 protein level in cytosol increased (*p* = 0.0039, *p* = 0.0013, and *p* = 0.0011 for 5, 20, and 50 ppm F^-^, respectively) (Figure 3D). In membrane fractions, the amounts of phospho-GluA1 subunits, as well as the phospho/total ratio, remained stable (Figure 3C,E).

**Figure 4:**
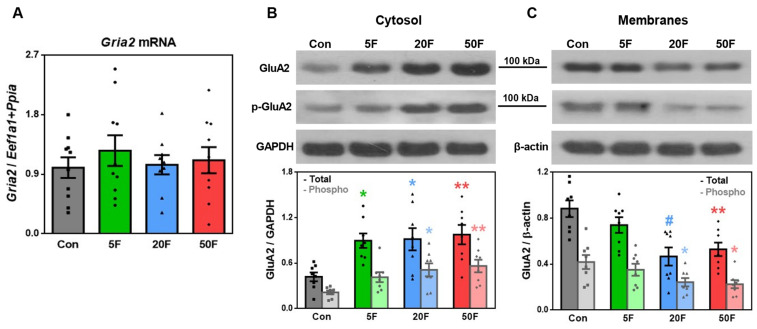
mRNA and protein levels of GluA2 subunits of AMPARs in the hippocampus of F^-^ poisoned rats. (**A**) Expression of *Gria2* gene in hippocampus of rats exposed to different F^-^ amounts (average values ± SEM, n = 10). (**B**,**C**) Alterations in the protein content of native and phosphorylated (Tyr869/Tyr873/Tyr876) forms of GluA2 subunits in the cytosol and membranes of hippocampal cells, respectively. Typical immunoblots and average values ± SEM (n = 8). * *p* < 0.05, ** *p* < 0.01, # *p* < 0.001 in comparison to controls.

The mRNA levels of the *Gria2* gene encoding GluA2 subunits of AMPARs did not differ between the hippocampi of rats who consumed excessive F^-^ amounts and that of control animals (Figure 4A). However, the pronounced changes were revealed at the protein level. Thus, the relative content of the GluA2 subunit increased in the cytosolic fraction of the hippocampus of F^-^-exposed animals (*p* = 0.0164, *p* = 0.0119 and *p* = 0.0045 vs. control for 5, 20 and 50 ppm F^-^, respectively), but decreased in membranes after consumption of 20 (*p* = 0.0007) and 50 (*p* = 0.0037) ppm F^-^ (Figure 4B,C). Similar alterations were observed for phosphorylated (Tyr869/Tyr873/Tyr876) form of GluA2 subunits at 20 (*p* = 0.0143 for cytosol and *p* = 0.0417 for membranes) and 50 (*p* = 0.0037 for cytosol and *p* = 0.0232 for membranes) ppm F^-^. However, the phospho-GluA2/total-GluA2 ratios did not change.

**Figure 5:**
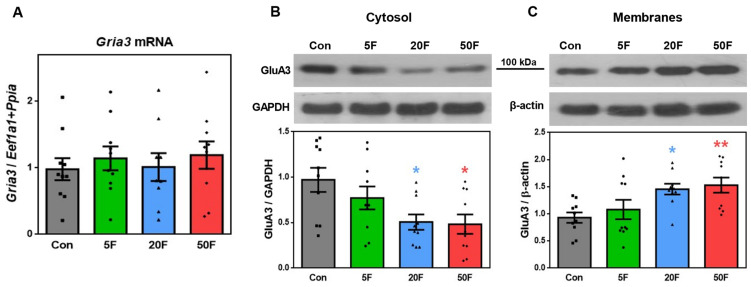
Effect of F^-^ on expression of GluA3 AMPARs subunits in rat hippocampus. (**A**) Expression of *Gria3* gene in hippocampus of animals consumed different F^-^ doses (averages ± SEM, n = 10). (**B**,**C**) Typical immunoblots and average values ± SEM for GluA3 protein levels in cytosol and membranes (n = 8). * *p* < 0.05, ** *p* < 0.01 in comparison to control.

The expression of the *Gria3* gene encoding GluA3 subunits of AMPA receptors was stable in the hippocampal tissues of rats from all groups, including those exposed to high F^-^ doses (Figure 5A). However, the protein expression level of GluA3 subunits declined in the cytosolic fraction of hippocampal cells after the impact of 20 and 50 ppm F^-^ (*p* = 0.0197 and *p* = 0.0135, respectively) but increased in membranes (*p* = 0.0242 and *p* = 0.0087) (Figure 5B,C).

Since many neuropathological conditions are accompanied by shifts in levels of different subunits composing AMPARs, we calculated the ratio between the expression of *Gria* genes and of GluA subunits composing Ca^2+^-impermeable and Ca^2+^-permeable receptors. This analysis did not reveal any changes in the *Gria1*/*Gria2* mRNA ratio in the hippocampal cells of rats consumed 5 and 20 ppm F^-^ but showed its statistically significant elevation at 50 ppm F^-^ (*p* = 0.0433) (Supplementary Figure S1A). The GluA1/GluA2 ratio for both native and phosphorylated forms of proteins remained unaltered in the cytosolic fraction (not shown) but considerably enhanced in membranes of animals from 20F (*p* = 0.0219 and *p* = 0.0040 for total and phosphorylated forms, respectively) and 50F (*p* = 0.0498 and *p* = 0.0136) groups (Supplementary Figure S1C). *Gria3*/*Gria2* gene expression ratio did not change as well (Supplementary Figure S1B), although GluA3/GluA2 ratio decreased in cytoplasm (*p* = 0.0070, *p* = 0.0005 and *p* = 0.0002 for 5, 20 and 50 ppm F^-^, respectively) (Supplementary Figure S1E), but increased in membrane fractions of cells after intake of 20 (*p* = 0.0003) and 50 (*p* = 0.0058) ppm F^-^ (Supplementary Figure S1F). Besides, chronic F^-^ poisoning led to a rise in the GluA3/GluA1 ratio in hippocampal membranes of rats given 20 and 50 ppm F^-^ (*p*= 0.0413 and *p* = 0.0081) (Supplementary Figure S1D).

### 2.4. Effect of Fluoride on Expression of Subunits Composing NMDA Receptors in Rat Hippocampus

**Figure 6:**
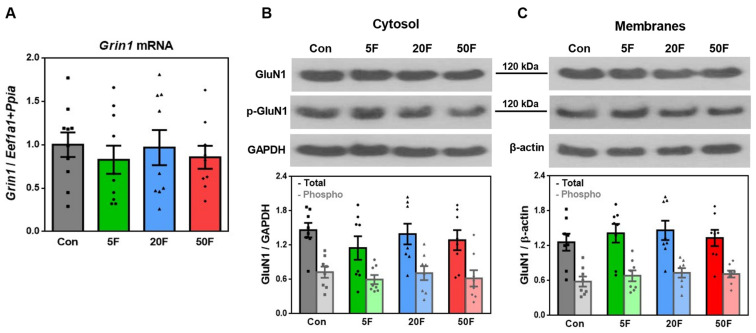
mRNA and protein levels of GluN1 subunits of NMDARs in hippocampal cells. (**A**) Relative expression of *Grin1* gene in the hippocampus of rats consumed different F^-^ doses normalized to that of a pair of reference genes (*Eef1a+Ppia*). Average values ± SEM (n = 10). (**B**,**C**) Protein expression of native and phosphorylated (Ser890) forms of GluN1 subunit in cytosolic and membrane fractions, respectively. Shown are typical immunoblots and average values ± SEM (n = 8).

The expression level of the *Grin1* gene encoding the GluN1 subunit of NMDA receptors did not respond to excessive F^-^ consumption by the animals (Figure 6A). Accordingly, increasing F^-^ concentrations did not change the subcellular distribution of GluN1 subunits. The relative content of both native and phosphorylated (Ser890) GluN1 forms in cytosolic and membrane fractions of hippocampal cells (Figure 6B,C), as well as phospho/total ratios, were stable as well.

**Figure 7:**
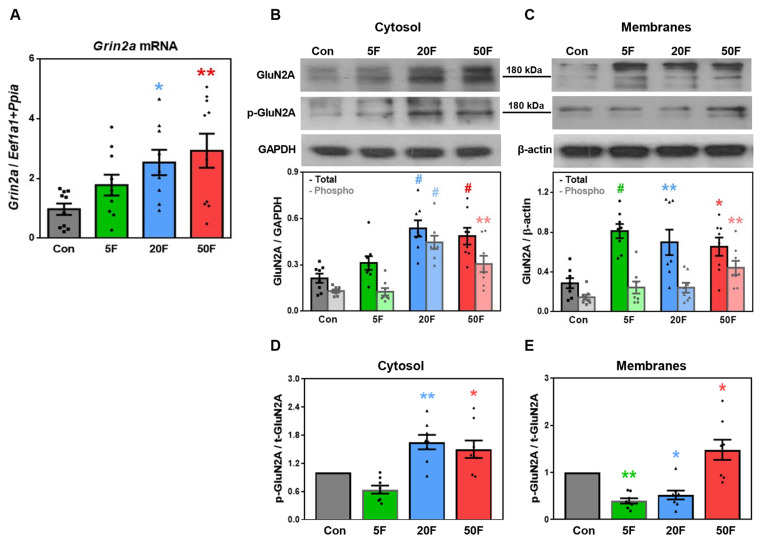
mRNA and protein content of GluN2A subunits of NMDARs in hippocampal cells. (**A**) Expression of *Grin2a* gene in hippocampus of rats consumed different F^-^ doses. Means ± SEM (n = 10). (**B**,**C**) Levels of native and phosphorylated (Tyr1246) forms of GluN2A subunit in the cytosolic and membrane fractions. Typical immunoblots and average values ± SEM (n = 8). (**D**,**E**) Phospho-GluN2A/total GluN2A ratios in cytosolic and membrane fractions (means ± SEM, n = 8). * *p* < 0.05, ** *p* < 0.01, # *p* < 0.001 compared to corresponding control.

The mRNA level of the *Grin2a* gene encoding GluN2A subunit of NMDA receptors significantly increased in the hippocampus of rats from 20F (*p* = 0.0331) and 50F (*p* = 0.0045) groups in comparison to control (Figure 7A). Accordingly, exposure of animals to 20 and 50 ppm F^-^ enhanced the expression of both total (*p* < 0.0001 and *p* = 0.0008) and phosphorylated (Tyr1246) (*p* < 0.0001 and *p* = 0.0059) forms of GluN2A subunits in the cytosol. The cytosolic phospho-GluN2A/GluN2A ratio increased as well (*p* = 0.0035 and *p* = 0.0300 for rats from the 20F and 50F groups, respectively) (Figure 7D). In membranes, the changes in total protein content occurred following intake of all three F^-^ doses (*p* = 0.0009, *p* = 0.0092, and *p* = 0.0214 for 5F, 20F, and 50F, respectively) (Figure 7B,C), while the level of phosphorylated form enhanced only in the cells of rats from 50F group (*p* = 0.0019). Phospho-GluN2A/total-GluN2A ratios increased in hippocampal cells of all animals consumed excessive F^-^ (*p* = 0.0042, *p* = 0.0268 and *p* = 0.0268 for 5, 20 and 50 ppm F^-^, respectively) (Figure 7E).

**Figure 8:**
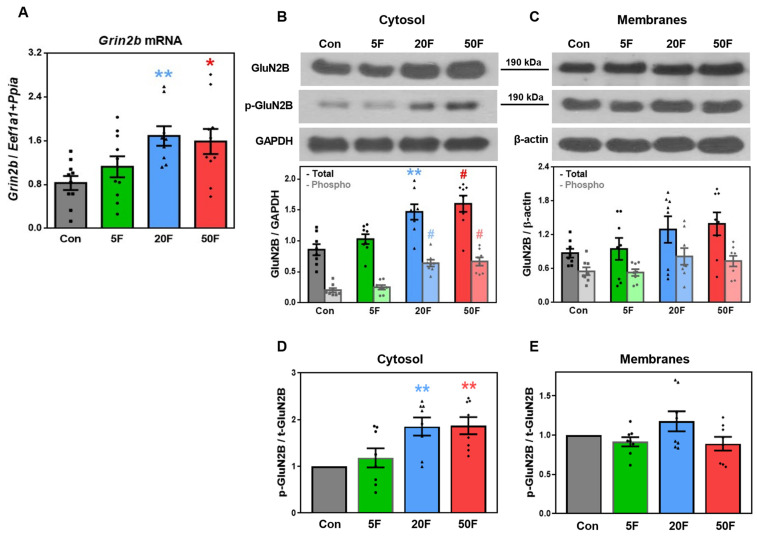
Expression of GluN2B subunits of NMDARs in hippocampal cells of F^-^-exposed rats at transcriptional and translational levels. (**A**) Levels of *Grin2b* gene mRNA (mean values ± SEM, n = 10). (**B**,**C**) Content of native and phosphorylated (Tyr1472) forms of GluN2B subunit in cytosolic and membrane fractions. Shown are typical immunoblots and average values ± SEM (n = 8). (**D**,**E**) Changes in phospho-GluN2B/total GluN2B ratios in cytosolic and membrane fractions (means ± SEM, n = 8). * *p* < 0.05, ** *p* < 0.01, # *p* < 0.001 compared to control.

The expression of the *Grin2b* gene encoding the GluN2B subunit of NMDA receptors was unchanged in the hippocampus of rats exposed to 5 ppm F^-^ but considerably increased in the cells of animals given 20 and 50 ppm F^-^ (*p* = 0.0084 and *p* = 0.0184 vs. control, respectively) (Figure 8A). These changes were accompanied by a rise of both total and phosphorylated (Tyr1472) forms of GluN2B subunits in the cytosol of hippocampal cells of rats given 20 (*p* = 0.0013 and *p* < 0.0001 for native and phospho-GluN2B, respectively) and 50 (*p* = 0.0001 for GluN2B and *p* < 0.0001 for phospho-GluN2B) ppm F^-^, as well as by phospho-GluN2B/total-GluN2B ratios (*p* = 0.0039 and *p* = 0.0032 for 20 and 50 ppm F^-^) (Figure 8B,D). No alterations were revealed in the membranes of F^-^-poisoned animals (Figure 8C,E).

The mRNA content of the *Grin3a* gene was comparable in hippocampal cells of control rats and animals treated with 5 and 20 ppm F^-^ but slightly increased in the cells of rats from the 50F group (*p* = 0.0364) (Supplementary Figure S2).

*Grin2a*/*Grin2b* expression ratios did not change in the hippocampus of rats exposed to excessive F^-^, while *Grin2a*/*Grin1* (*p* = 0.0219) and *Grin2b*/*Grin1* (*p* = 0.0442) ratios increased at 20 ppm F^-^ (Supplementary Figure S3A,C,E). The ratio between native GluN2A/GluN2B subunits in the cytosol was unaltered as but significantly increased in membranes of hippocampal cells from all F^-^-poisoned rats (*p* = 0.0014, *p* = 0.0198 and *p* = 0.0465 for 5F, 20F and 50F, respectively), while that of phosphorylated forms enhanced in hippocampi of animals given 50 ppm F^-^ (*p* = 0.0006) (Supplementary Figure S3B). In contrast, the changes in the GluN2A/GluN1 ratio were observed predominantly in the cytosolic fraction of cells obtained from rats given 20 (*p* = 0.0217 and *p* = 0.0228 for native and phosphorylated forms, respectively) and 50 (*p* = 0.0490 for total and *p* = 0.0426 for phospho-forms) ppm F^-^ (Supplementary Figure S3D,F). The shift in GluN2B/GluN1 ratio was statistically significant in the cytosol of hippocampal cells of rats exposed to 20 (*p* = 0.0015 for phospho-form) and 50 (*p* = 0.0402 and *p* = 0.0007 for total and phospho-forms, respectively) ppm F^-^.

## 3. Discussion

The results of the present study show a complicated picture of alterations in the expression of different subunits composing AMPA and NMDA glutamate receptors in hippocampus of F^-^-exposed rats at both transcriptional and translational levels (Figure 3, Figure 4, Figure 5, Figure 6, Figure 7 and Figure 8).

Changes in synaptic AMPAR number and subunit composition are the primary mechanisms underlying synaptic plasticity and cognitive capacities [28,29,30]. On the other hand, subcellular AMPAR redistribution, such as the replacement of Ca^2+^-impermeable GluA2-containing AMPARs with Ca^2+^-permeable GluA1-dominant AMPARs at synapses, is observed in many neuropathological conditions [30]. Thus, endocytosis of GluA2-containing AMPARs and facilitated delivery of GluA2-lacking receptors to synaptic sites was found in the spinal cord of rats with peripheral nerve injury [31] and diabetic neuropathy [32] or in hippocampal neurons after ischemic insult [33]. Internalization of AMPARs associated with LTD (long-term depression) and synaptic failure has been linked with Alzheimer’s disease (AD) [34]. Disturbed trafficking and reduced AMPAR number were observed at synaptic and extrasynaptic membranes of different types of neurons in P301S tau and APP/PS1 transgenic mouse AD models [35,36]. Stress-induced behavior changes were associated with the insertion of Ca^2+^-permeable AMPARs in the amygdala [37], while decreased AMPAR levels were revealed in different brain regions of schizophrenic patients [38]. The dysfunction of GluA3-containing AMPARs at least partially underlies amyloid-β-triggered memory deficits [39], mild cognitive decline [40], and aggressive behavior [41].

The negative impact of F^-^ on cellular AMPARs has been reported in a few early works. Depressed LTP in hippocampal microglial cells following exposure of rats to 120 ppm F^-^ for 12 weeks was associated with decreased content of GluA2 subunits [42]. The level of *Gria2* mRNA was reduced in the brains of mouse pups exposed to 25–100 ppm NaF prenatally and during lactation, although no changes were observed for GluA1 [43]. In contrast, total cellular levels of GluA1 and GluA2 AMPAR subunits in mice hippocampus and cortex did not respond to combined prenatal/P90 exposure to 25 ppm F^-^ [21]. In this study, we observed considerable alterations in the levels of different AMPAR subunits in subcellular fractions. An increased GluA2 protein content in the cytosol probably indicates intracellular retention of this subunit (Figure 4). In contrast, changes in the location of GluA3 subunits can be linked with membrane insertion (Figure 5). Such subcellular redistribution of GluA2 and GluA3 at the background of stable GluA1 expression (Figure 3) suggests the shift in the ratio between Ca^2+^-permeable and Ca^2+^-impermeable AMPARs in membranes (Supplementary Figure S1) probably leading to the prevalence of Ca^2+^-permeable AMPARs at synaptic sites. Moreover, such changes are typical for neurodegenerative processes. However, the stable expression of corresponding *Gria* genes indicates that all alterations in AMPAR levels in the rat hippocampus are provided exclusively by their post-translational modifications.

Phosphorylation of GluA subunits is another mechanism regulating AMPAR biophysical properties and functions, as well as their trafficking to synapses or endocytosis [25,44,45,46]. The study on mice with mutations at GluA1 Ser845 and Ser831 confirmed an important role of phosphorylation in learning and motivated behavior [47]. Ser845-GluA1 phosphorylation is believed to control the recruitment of this subunit to membranes for synaptic insertion or removal from synapses during synaptic plasticity, while Tyr876-GluA2 phosphorylation prevents endocytosis. Acute stress, declined cognitive capacities, and depressive disorders are accompanied by phosphorylation of both GluA1 (Ser845 and Ser831) and GluA2 (Ser880) subunits in different brain regions [48,49,50]. The altered ratio between native and phosphorylated GluA1 subunits underlie memory impairment in the APP/PS1 mouse AD model [51]. In our work, an enhanced phospho/total GluA1 ratio revealed in the cytoplasm of hippocampal cells of F^-^-poisoned animals point to activation of this subunit (Figure 3). Ser845-GluA1 phosphorylation might facilitate the insertion of GluA1-containing AMPARs to postsynaptic density or indicate the formation of a reserve pool of these molecules. An increased level of phosphorylated GluA2 subunits in the cytosol but its declined amount in membranes with the stable phospho/total GluA2 ratio is probably entirely linked with their subcellular redistribution (Figure 4). Overall, such changes suggest an impaired intracellular GluA1 and GluA2 subunit trafficking, contributing to an altered ratio of various GluA subunits in membrane AMPARs.

Similar to AMPARs, an overwhelming body of evidence centered on NMDARs as inductors of synaptic plasticity, with the expression of GluN1 and GluN2A subunits increasing during memory formation and consolidation [52,53,54]. Despite this favorable role, the changes in the GluN2A/GluN2B ratio can induce excitotoxicity and implicate a wide range of neurological disorders [55]. Increased GluN2A/GluN2B ratio in amygdala neurons of transgenic mice before fear learning significantly disrupted long-term memory consolidation [56]. Changed GluN2A/GluN2B ratio following the knockdown of the *Grin2a* gene led to impaired contextual fear-conditioning memory and increased seizure susceptibility [57]. Methamphetamine-induced schizophrenia in rats was accompanied by decreased expression of *Grin* genes in different brain regions [58]. Mutations in the *GRIN2B* gene are associated with intellectual disability, developmental delay, motor impairments, autism spectrum disorder, and epilepsy in humans [59]. The changed ratio between GluN2A- and GluN2B-containing NMDARs, their allosteric modulations, disturbed trafficking, or impaired interaction with AMPARs are well-acknowledged events in epileptogenesis [60,61,62]. On the other hand, a few works [63,64,65] suggested the neuroprotective function of hippocampal astrocytic NMDARs since pharmacological antagonism of GluN2A and GluN2B or knockdown of *Grin2a* gene exacerbates β-amyloid (Aβ)-induced synaptotoxicity and cognitive decline.

The data describing an F^-^ impact on NMDAR functions are controversial. Using three experimental systems (brains of adult rats given 50 ppm F^-^ for 6 months, their offspring sacrificed during early postnatal life, and primary cultured hippocampal neurons of neonatal rats treated with 5 and 50 ppm F^-^ for 48 h), Wei et al. [66] showed that gene and protein expression of GluN1 and GluN2B subunits enhanced, GluN2A did not change, and GluN3A decreased. A similar increase of NMDAR1 mRNAs and protein was described in the hippocampus of rat pups exposed to 150 mg/L NaF prenatally and until PND28 [20]. Upregulation of GluN2A and downregulation of GluN2B protein expression was observed in the hippocampus of mice exposed to 25 ppm F^-^ prenatally/up to P90 [21]. In contrast, down-regulation of NMDAR mRNA or protein was reported in the brains of offspring mice treated with 50–100 ppm F^-^ for 90 days [67]. Microglia-induced neuroinflammation in the hippocampus of rats given 120 ppm F^-^ for 12 weeks was associated with a decline in NMDA2B protein [42].

In our study, in contrast to AMPARs, F^-^-induced changes in the expression of NMDARs subunits in rat hippocampus occurred at both transcriptional and translational levels (Figure 6, Figure 7 and Figure 8). Overexpression of *Grin2a* and *Grin2b* genes suggests enhanced synthesis, while increased phosphorylation of GluN2A and GluN2B subunits can be associated with their intensive trafficking. However, elevated *Grin2a/Grin1* and *Grin2b/Grin1* ratios, cytosolic GluN2A/GluN1 and GluN2B/GluN1 ratios, and membrane GluN2A/GluN2B ratio (Supplementary Figure S3) indicate the severe disturbances in synaptic processes in response to F^-^ exposure. In spite of both GluN2A and GluN2B subunits being upregulated, an increase in membrane GluN2A/GluN2B ratio might be linked with the insertion of newly synthesized GluN2A-dominant NMDARs to membranes and internalization of GluN2B-containing receptors. Increased phosphorylation of GluN2B (Tyr1472) subunits in the cytosol can facilitate their association with postsynaptic density protein PSD-95 or stabilize the extrasynaptic location of GluN2-containing NMDARs. However, stable expression of obligatory GluN1 subunit at the background of increased expression of GluN2A and GluN2B subunits might be associated with altered expression of other minor NMDAR subunits, which were not examined in our study.

Overall, we assume that changes in the ratio between Ca^2+^-permeable and Ca^2+^-impermeable AMPARs subunits and between GluN2A and GluN2B subunits in membrane NMDARs induce excitotoxicity and at least partially underly an impairment in cognitive capacities of F^-^-poisoned animals (Figure 1 and Figure 2). Fluoride ions are known to form stable complexes with metal ions such as Mg^2+^, Mn^2+^, Al^3+,^ and Fe^3+^ [68], which theoretically allows them to interact with enzymes possessing these metals in active centers. Thus, F^-^ directly binds with the structural and catalytic Mg^2+^ in glycolytic metalloenzyme enolase [69]. Besides, F^-^ is a potent inhibitor of protein phosphatases [70]. Although these effects have been shown in vitro and the doses of F^-^ sufficient for triggering such reactions in vivo are not established, we assume that effects of F^-^ on NMDARs in the rat hippocampus are associated with its binding to Mg^2+^, while increased receptors phosphorylation is at least partially due to impaired dephosphorylation. Hyperactivation of AMPARs and NMDARs can induce an excessive Ca^2+^ entrance to the cells and activate Ca^2+^-dependent enzymes, including protease calpain, which destroys cytoskeletal structures, leading to pathological changes in neurons [15,16]. The functional activity of calpain is closely associated with the stimulation of NMDARs [71]. On the other hand, since GluN2A subunits are believed to have a protective role in brain cells, such changes can indicate a compensatory mechanism necessary for alleviating the rate of neuron death under the influence of F^-^.

It is interesting that altered expression profiles of subunits composing AMPARs and NMDARs were pronounced in the hippocampus of rats given 20 and 50 ppm F^-^, whereas the decline in cognitive capacities was also evident for animals given the lowest F^-^ dose (5 ppm). This phenomenon suggests the existence of other cellular processes providing a negative F^-^ impact on brain functions. Recently, impaired neurogenesis and death of hippocampal neurons under F^-^ influence was linked with activation of Notch1 (Neurogenic locus notch homolog protein 1) signaling, dysfunction of SIK2-CRTC1 (Salt inducible kinase 2-CREB-regulated transcription coactivator 1) signaling, and declined synthesis of BDNF (brain-derived neurotrophic factor) and VGF (nerve growth factor inducible) [16,72,73].

The strength of our study is the application of relatively low F^-^ doses, which yielded plasma F^-^ content comparable to that registered in the serum of humans from regions of endemic fluorosis, taking into account faster F^-^ clearance in rats. The long period of F^-^ exposure, which led to the development of stable fluorosis, also allowed us to repeat the conditions common in the areas with high environmental F^-^ content. Next, this study is the first to comprehensively evaluate the changes in expression of all major AMPARs and NMDARs in rat hippocampus following long-term F^-^ intake. However, our work has some limitations. First, as for all in vivo studies, the methods applied by us cannot distinguish between neurons and glial cells, between synaptic and extra-synaptic receptors, between their trafficking to membranes or endocytosis, so we cannot evaluate the number of receptors directly implicated in excitotoxic F^-^ effects, as well as cannot say which of the processes of synaptic plasticity (LTP or LTD) is disturbed. Next, the present study did not explore the possible compensatory mechanisms that can be activated in response to hyperactivation of AMPARs and NMDARs to alleviate excitotoxicity.

## 4. Materials and Methods

### 4.1. Animals

Forty male conventional healthy Wistar rats were procured from the Animal Facility of Sechenov Institute and maintained under standard conditions (temperature of 22–25 °C, 12 h light/12 h dark cycle, food and water ad libitum). Low F^-^ natural-ingredients diet was manufactured following recommendations of Nutrient Requirements of Laboratory Animals [74]. All procedures were in accordance with the Animal Welfare Act (2006) and approved by the Bioethics Committee of Sechenov Institute of Evolutionary Physiology and Biochemistry (Protocol # 9/2021 of 24 September 2021).

At the age of 6 weeks, the animals were randomly assigned into four groups. Control rats were given regular tap water with a natural F^-^ content of 0.4 ppm provided by the local water supplier. Animals from other groups received the same water supplemented with 5, 20, and 50 ppm F^-^ (as NaF) for 12 months. This treatment period was sufficient for rats to develop stable dental fluorosis, as was established in our previous work [16]. The doses of F^-^ were comparable to those existing in the water available for humans in the regions of endemic fluorosis, taking into account that the rate of F^-^ clearance in rats is higher than in humans. For instance, the F^-^ content of 1.72 µM in the serum of rats given 22.6 ppm F^-^ [75] was similar to the F^-^ concentration in the plasma of humans consuming 2–3 ppm F^-^ [76,77].

The development of fluorosis was evaluated by the changes in animal teeth enamel, as described earlier [16]. While the incisors of control rats were yellowish and matte, the teeth of animals consumed 50 ppm F^-^ became “glass-like” with white stains, which indicates impaired enamel mineralization typical for dental fluorosis in rodents. The values of Dean’s fluorosis index calculated for the rats from control and F^-^-poisoned groups are shown in Supplementary Table S1. In the group given 5 ppm F^-^, less than half of the animals had normal teeth, while the majority of rats consumed 20 and 50 ppm F^-^ developed moderate and severe degrees of fluorosis, respectively.

### 4.2. F^-^ Determination

F^-^ content in the whole brain was evaluated in a separate cohort of animals exposed to background F^-^ content (0.4 ppm), 5 and 20 ppm F^-^. One hemisphere of the brain was homogenized in phosphate-buffered solution (pH 7.4) at 4 °C. The homogenates were centrifuged for 15 min at 22,000× *g* to obtain pure supernatant. F^-^ concentrations in the samples were determined using an F^-^-specific electrode (Elit-221, Akvilon, Russia) and AgCl_3_ reference electrode connected to potentiometer I-500 (Akvilon, Russia). Calibration curves were created using the standards with known F^-^ concentrations in water buffered with TISAB II (pH 5.15) (AppliChem, Darmstadt, Germany). Aliquots of brain homogenates were mixed with an equal volume of TISAB II buffer. The brain samples were prepared under the same conditions as the standards. Brain F^-^ content was expressed as µg of fluoride/g of tissue.

### 4.3. Examination of Cognitive Capacities

The efficiency of hippocampus-dependent spatial and non-spatial memory and learning was evaluated by three behavior tests widely used in rodents. The rats were subjected to behavior experiments within the last four weeks of F^-^ exposure. All test series were recorded by Any-maze behavioral Video Tracking System 6.36 software and analyzed using Any-maze 4.48 program (Stoelting Co., Wood Dale, IL, USA).

#### 4.3.1. Novel Object Recognition Test (NOR)

The formation of short-term and long-term non-spatial memory was evaluated using the NOR test. Before testing, the rats were habituated to an experimental cage by being allowed to explore it for 5 min in the absence of objects. The next day, a training session was performed first. The rats were placed at the center of the cage in front of two identical objects and allowed to examine them for 5 min. The formation of short-term memory was examined 1 h after training and the efficiency of long-term memory 24 h after. During both testing sessions, one of the familiar objects was replaced with a new one. All objects were odorless glasses of different shapes and colors but with similar volumes and heights. The recognition was considered successful if the rats touched the objects with their noses or hands. The objects were cleaned with 70% ethanol after each rat to remove the smell of the previous animal. The results of both testing sessions are presented as discrimination index: DI = (novel object)/(novel object + familiar object) × 100% [78].

#### 4.3.2. Y-Maze Test

The Y-maze test was performed a week after the NOR test and used to evaluate spatial working memory. The maze was made of black plastic and contained three arms (50 × 10 × 30 cm) positioned at a constant angle. Each rat was placed at the center of the maze and allowed to freely explore it for 8 min. The number of individual arm entries and their sequence were scored by an observer in a triplet set, with successful entry defined as all four animal legs inside the arm. The percentage of spontaneous alternation behavior was calculated as the ratio of actual to possible alternations: C_alt_ = N_alt_/(N_t_ − 2) × 100%, where N_alt_—number of alternations, N_t_—number of total arms entries [79].

#### 4.3.3. Morris Water Maze Test

Spatial learning and formation of memory were evaluated by the Morris water maze test [80] a week after the Y-maze test. The experiments were performed in a circular plastic pool (TS1004-M2G, Water maze test, OpenScience, Moscow, Russia) filled with opaque water (temperature of 22–24 °C), which was changed every day. The pool was divided into four quadrants (north, south, east, and west) used as the starting points. Before the test series, the animals were habituated to water by swimming for 60 s. The next day, the escape platform was fixed in the geometric center of the northwest quadrant, 1 cm above the water, and four objects of various shapes were placed on the walls of the pool to help animals learn about the environment. The rats were allowed to find the platform and stay on it for 15 s. During the next four consecutive days, the animals were subjected to hidden-platform acquisition tests from different sides. The rats were allowed to find the platform submerged 1 cm below water for 60 s. The time required to locate the platform (escape latency) was established. If the rat failed to find the platform within testing time, it was guided there by the researcher, and escape latency was fixed at 60 s. After finding the platform, the rats were allowed to rest on it for 15 s. Each day, four consecutive swimming attempts from different starting points with a rest interval of 15 s were performed. The swimming time, pathway, and speed were analyzed. The second stage of the test (spatial probe) was performed on day 5. The platform was removed, and the rats were placed into the pool facing the wall in the quadrant opposite the original position of the platform. The swimming pathway was recorded for 60 s. The time of the first entry to the target quadrant and a few other parameters have been analyzed in the Any-maze program.

### 4.4. mRNA Extraction and cDNA Synthesis

In two weeks after the end of behavior tests, the rats were anesthetized with Zoletil-100 by intraperitoneal injection (50 mg/kg BW) and sacrificed by dissecting the abdominal aorta. The brains were removed and placed in ice-cold conditions. The hippocampi from one hemisphere were isolated and incubated in an Intact RNA reagent (Eurogen, Moscow, Russia) for 24 h at 4 °C to stabilize RNA. Then, the samples were homogenized in the appropriate volume of monophasic aqueous ExtractRNA reagent (Eurogen, Moscow, Russia) according to the manufacturer’s instructions. Total RNA concentration (absorption at 260 nm) and purity (260/280 nm absorption ratio) were evaluated using a CLARIOstar Plus multimodal reader (BMG LABTECH, Ortenberg, Germany). The absorption ratio at 260/280 nm exceeded 1.8 in all samples. Total RNA (1 μg) was reverse transcribed into cDNA using an MMLV RT kit (Eurogen, Moscow, Russia). The reaction mixture was incubated for 5 min at 25 °C, 60 min at 42 °C, and 5 min at 70 °C. cDNA was diluted 10-fold before the PCR step. The primers were provided by Eurogen (Moscow, Russia) (Supplementary Table S2). The melting temperature was optimized using the Primer Blast tool (http://www.ncbi.nlm.nih.gov/tools/primer-blast, accessed on 1 October 2024). The product size was 70–250 base pairs, and GC content was 40–60%.

### 4.5. Quantitative Real-Time PCR

RT-qPCR was performed on a C1000 Touch Thermal Cycler equipped with the CFX96 detection system (Bio-Rad, Hercules, CA, USA). The reactions were carried out using qPCRmix-HS SYBR Master Mix (Eurogen, Moscow, Russia). The mixture contained 17 µL of dH_2_O, 2 µL of direct and reverse primer (500 nM), 1 µL of cDNA, and 5 µL of qPCRmix-HS SYBR. The cycling protocol included initial denaturation at 95 °C for 5 min followed by 40 amplification programs, each consisting of a denaturation step at 95 °C for 5 s, an annealing step at 57–63 °C for 10 s, and an extension step at 72 °C for 30 s. The specificity of amplification was verified by analysis of melting curves.

qPCR curves were analyzed using CFX Manager version 3.1 software (Bio-Rad Laboratories, Inc., Hercules, CA, USA). The expression of target genes was normalized to that of the *Eef1a1/Ppia* pair of reference genes, showing stable expression in our previous work [81]. The threshold cycle (Ct) values for target and reference genes were less than 40 for all reactions. The relative expression of genes was estimated by the comparative 2^−ΔΔCt^ method, where ΔCt is the difference between studied and reference genes [82]. The average Ct and standard deviation (SD) values for each qPCR product were calculated from three individual repeats.

### 4.6. Western Blotting Analysis

The hippocampi from the second hemisphere were frozen at −80 °C until processing. The subcellular fractions enriched with cytosolic and membrane proteins (further referred to as cytosol and membranes, respectively) were obtained using a slightly modified method of detergent extraction recommended for Mem-PER Plus Membrane Protein Extraction kit (#89842; ThermoFisher Scientific, Waltham, MA, USA). The hippocampi were minced with permeabilization buffer supplemented with inhibitors of proteases (Sigma-Aldrich P8340, Merck, Darmstadt, Germany) and phosphatases (1 mM Na_3_VO_4_ 2 H_2_O and 1 mM Na_2_-EDTA). Non-homogenized material was sedimented by centrifugation at 1000× *g* for 15 min and 4 °C. The supernatant was centrifuged at 11,000× *g* for 15 min and 4 °C, then the aliquots of the second supernatant were centrifuged at 25,000× *g* for 15 min and 4 °C to obtain the fraction enriched with cytosolic proteins, and the final pellet was solubilized to obtain the fraction containing membrane and membrane-associated proteins. The purity of obtained fractions was examined by immunoblotting with membrane proteins Na-K-ATPase, postsynaptic density protein PDS95, mitochondrial membrane protein VDAC (voltage-dependent anion channel), cytosolic protein GAPDH and cytoskeletal protein β-actin. For this purpose, an equal amount of total protein (30 µg) from all fractions was loaded into wells. As seen in Supplementary Figure S4, the fractions enriched with cytosolic and membrane proteins were reasonably homogenous and contained only small amounts of the proteins from other subcellular fractions.

The samples were frozen at –80 °C until use. The total protein content in the probes was determined by the Lowry method. The samples were solubilized in Laemmli buffer, then 60 or 100 µg/well of cytosolic and 30 µg/well of membrane proteins were separated by SDS–PAGE in 10% polyacrylamide gels and transferred to nitrocellulose membranes. Non-specific membrane binding was blocked for 1 h at room temperature with a 5% skim milk solution. Then the membranes were incubated overnight with primary antibodies against GluA1 (1:750), phospho-GluA1 (1:750), GluA2 (1:750), phospho-GluA2 (1:750), GluA3 (1:750), GluN1 (1:500), phospho-GluN1 (1:500), (1:200), GluN2A (1:500), phospho-GluN2A (1:200), GluN2B (1:500) and phospho-GluN2B (1:200), followed by 1-h treatment with secondary HRP-conjugated antibodies (1:1000). Immunostained proteins were visualized by exposure with ECL detection system. To control protein loading, the membranes were stripped by two rounds of 30-min incubation with an acidic solution containing 25 mM glycine and 1% SDS (pH 2.0), followed by 10-min TBS washing and re-probed with antibodies against GAPDH for cytosol or β-actin for membranes (1:1000) followed by secondary HRP-conjugated antibodies. Relative protein expression was evaluated by densitometric analysis using the ImageJ program (NIH, Bethesda, MD, USA). The optical densities of target proteins were normalized to that of reference proteins.

Rabbit antibodies to GluA1 (#13185), phospho-GluA1 (#8084), GluA2 (#5306), phospho-GluA2 (#3921), GluA3 (#4676), GluN1 (#5704), phospho-GluN1 (#3381), GluN2A (#4205), phospho-GluN2A (#4206), GluN2B (#4207) and phospho-GluN2B (#4208) were purchased from Cell Signaling Technology (Danvers, MA, USA). Mouse monoclonal antibodies against GAPDH (sc-32233) and β-actin (sc-517582) were obtained from Santa Cruz Biotechnology. Secondary antibodies and chemiluminescent ECL systems were purchased from Cytiva (Marlborough, MA, USA), and X-ray film, developer, and fixer solutions were purchased from Agfa CEA (Mortsel, Belgium).

### 4.7. Statistics

The statistical analysis was performed using GraphPad Prism 6 software (San Diego, CA, USA). Shapiro-Wilk normality test was applied to check for the Gaussian distribution of the samples. The results of the Y-maze test were evaluated by the Mann-Whitney U-test. The parameters obtained in the MWN acquisition test, such as mean latencies and distance, were analyzed by two-way repeated measures (RM) ANOVA. All other data were evaluated by one-way ANOVA followed by Bonferroni multiple comparison test. The data are presented as mean ± SEM for parametric results and boxplots for non-parametric data. The differences were considered statistically significant at *p* < 0.05.

## 5. Conclusions

The results of our study revealed that the cognitive decline of F^-^-poisoned rats is accompanied by the changes in expression of a few subunits composing AMPA and NMDA receptors in hippocampal cells. The major findings are the prevalence of Ca^2+^-permeable GluA1- and GluA3-containing AMPA receptors in membranes, internalization of Ca^2+^-impermeable GluA2-containing AMPARs, upregulation of *Grin2–3* genes, and shift in the ratio between GluN2A and GluN2B subunits composing NMDA receptors. All these phenomena might indicate disturbed synaptic processes and are typical for neurological disorders.

## Figures and Tables

**Table 1:** The parameters of swimming obtained in MWM test during the spatial probe trial.

	Control	5F	20F	50F
Time to first entry to target quadrant (s)	5.5 ± 0.6	7.6 ± 0.9 *	8.8 ± 1.0 *	8.1 ± 0.9 *
Time spent in target quadrant (s)	17.8 ± 0.9	15.9 ± 1.7	14.9 ± 1.1 *	16.8 ± 1.6
Total distance (m)	7.1 ± 0.6	5.8 ± 0.6	5.7 ± 0.6	6.9 ± 0.7
Mean speed (m/s)	0.12 ± 0.01	0.10 ± 0.01	0.09 ± 0.01	0.12 ± 0.01
Distance in target quadrant (m)	1.6 ± 0.1	1.0 ± 0.1 **	1.2 ± 0.2 *	1.3 ± 0.2 *
Distance in opposite zone (m)	1.3 ± 0.2	1.7 ± 0.1	2.1 ± 0.3 *	2.6 ± 0.3 **
Number of entries to target quadrant	4.5 ± 0.4	3.3 ± 0.3 *	4.2 ± 0.5	4.5 ± 0.5

Comment: Target quadrant—quadrant in which the hidden platform was placed during the first stage of the test (1–4 days). Opposite zone—quadrant not possessing borders with the target zone. Average values *±* SEM are shown, * *p* < 0.05, ** *p* < 0.01 compared to control (n = 10).

## Data Availability

The data generated and analyzed during the current study will be available from the corresponding authors upon reasonable request.

## Supplementary Materials

The following supporting information can be downloaded at: https://www.mdpi.com/article/10.3390/ijms252111796/s1.
