# Supplementary material for: AMPA and NMDA Receptors in Hippocampus of Rats with Fluoride-Induced Cognitive Decline
Source: Int J Mol Sci. 2024 Nov 2;25(21):11796. doi: 10.3390/ijms252111796 (PMC11546234; doi:10.3390/ijms252111796)
Supplement: Supplementary file 1 [file ijms-25-11796-s001.zip › ijms-3253584-supplementary.pdf]

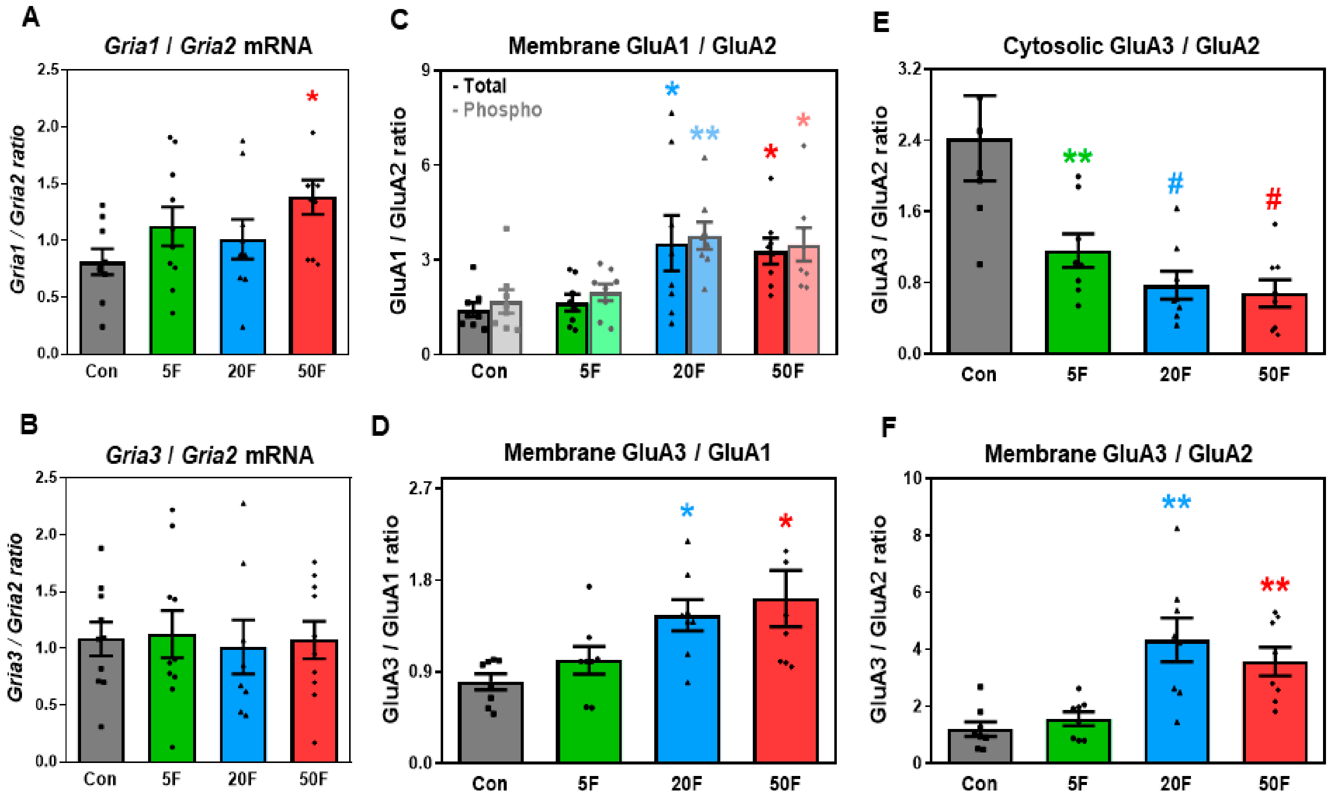

Supplementary Figure S1. The calculated ratios between expression levels of *Gria1* and *Gria2* genes (**A**); *Gria3* and *Gria2* genes (**B**) (average values ± SEM, n=10). The ratios between protein content of GluA1 and GluA2 subunits in membranes (**C**); GluA3 and GluA1 subunits in membranes (**D**); GluA3 and GluA2 subunits in cytosol (**E**) and membranes (**F**) (average values ± SEM, n=8). One-way ANOVA followed by Bonferroni multiple comparison test. * p < 0.05, ** p < 0.01, # p < 0.001 in comparison to control.

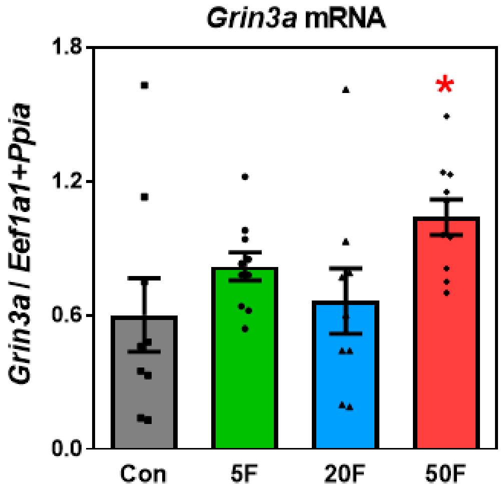

Supplementary Figure S2. Relative expression of *Grin3a gene* in the hippocampal cells of F^--exposed rats normalized to that of reference genes. Mean values ± SEM (n=10) are

presented. One-way ANOVA with Bonferroni post-hoc test. * p < 0.05 in comparison to control.

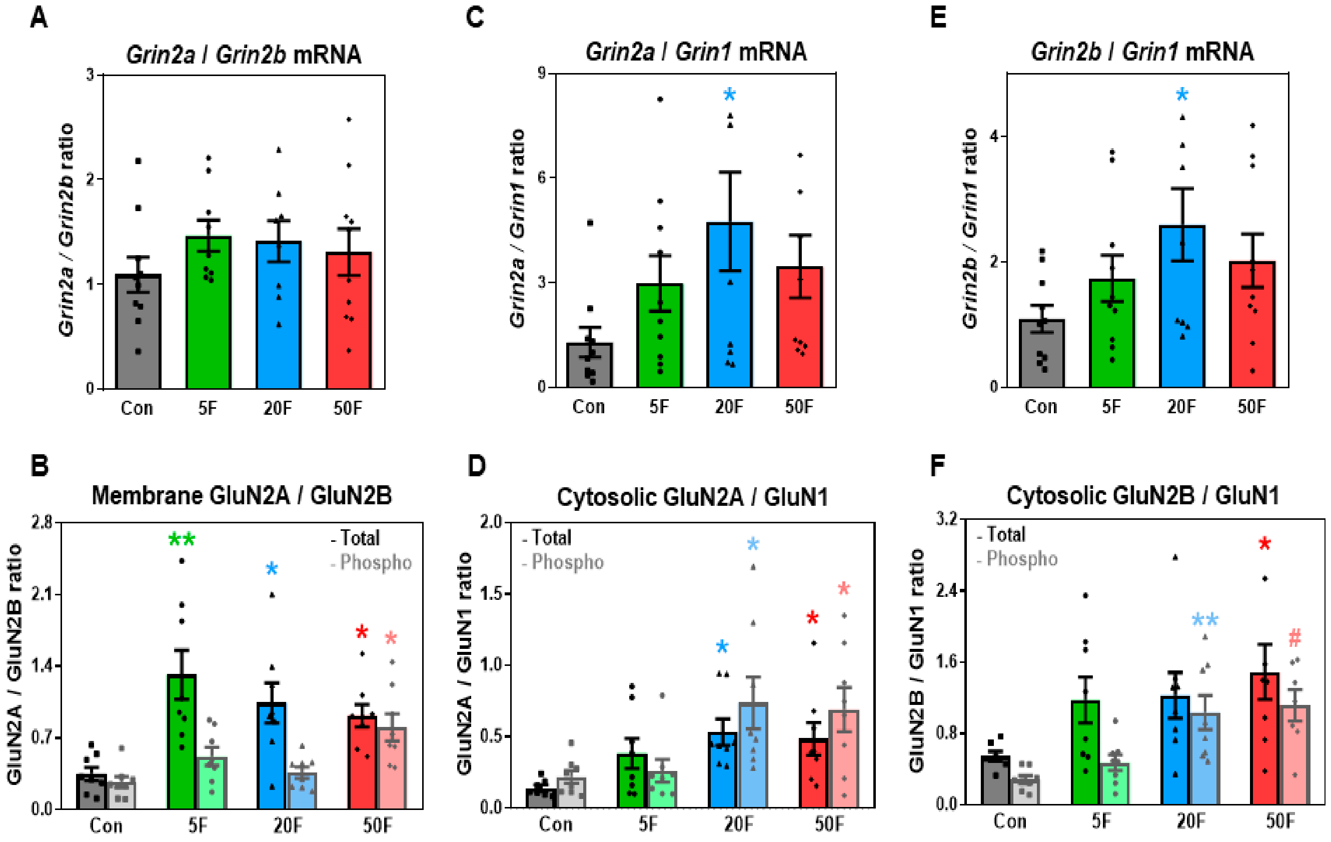

Supplementary Figure S3. The calculated ratios between expression levels of *Grin2a* and *Grin2b* genes (**A**) and between protein content of GluN2A and GluN2B subunits in membranes (**B)** of hippocampal cells; between *Grin2a* and *Grin1* genes (**C**) and GluN2A and GluN1 subunits in cytosol (**D**); between *Grin2b* and *Grin1* genes (**E**) and GluN2B and GluN1 subunits in cytosol (**F**). Presented are average values ± SEM, n=10 for mRNA levels, n=8 for protein content. One-way ANOVA followed by Bonferroni multiple comparison test. * p < 0.05, ** p < 0.01, # < 0.001 in comparison to control.

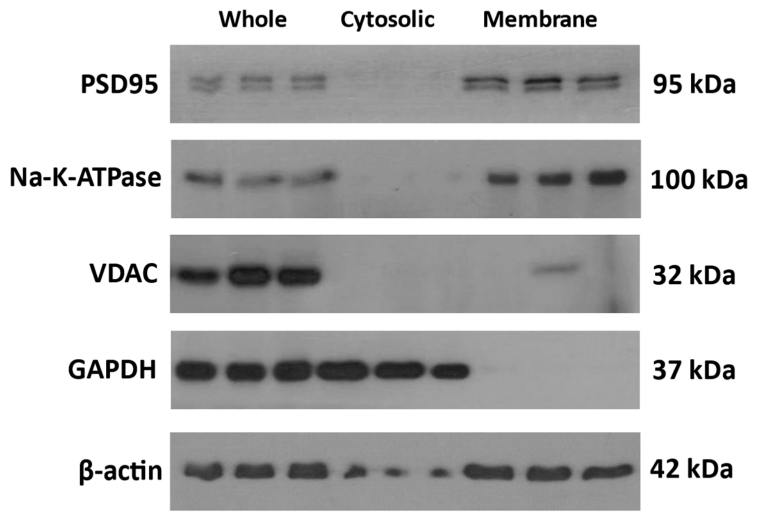

Supplementary Figure S4. Expression of cytoplasmic membrane proteins PSD95 and Na-K-ATPase, mitochondrial protein VDAC, cytosolic protein GAPDH and cytoskeletal protein β-actin in whole cell lysates and subcellular fractions enriched with cytoskeletal and membrane proteins. The presented results are typical examples of immunoreactivity of a few randomly chosen samples. Total protein content in the samples is 30 μg/well.

Supplementary Table S1. The degree of teeth fluorosis in rats consumed different F^- doses according to Dean's scale.

Group	Normal, %	Mild, %	Moderate, %	Severe, %
**Control (n=10)**	100	0	0	0
**5F (n=10)**	47	40	13	0
**20F (n=10)**	0	13	60	27
**50F (n=10)**	0	7	27	66

The percent of animals with different degree of teeth fluorosis relative to total number of rats in each experimental group.

Supplementary Table S2. Details of the primers used in the study.

Target gene/Amplicon length	Accession	Sequence/GC content	T(°C), time (s)	Amplification efficiency (%)/Correlation coefficient (R2)
Rattus norvegicus glutamate ionotropic receptor NMDA type subunit 1 (Grin1)/ 208 bp	NM_017010.2	Forward: TCTGTCCGCAGCCACAATAG Reverse: GGCATCACCACTGAATACCG	55, 30	100.73/0.994
Rattus norvegicus glutamate ionotropic receptor NMDA type subunit 2A (Grin2a)/ 178 bp	NM_012573.4	Forward: GCTCTAAGCCTCACCAGCAA Reverse: TGATACACCCCAAACCAGGC	57, 30	97.79/0.995
Rattus norvegicus glutamate ionotropic receptor NMDA	NM_012574.1	Forward: AGGAACCAGGCTACATCAAAAA Reverse: TAGTGATCCCACTGCCATGTAG	55, 20	94.72/0.997

type subunit 2B (Grin2b)/ 197 bp				
Rattus norvegicus glutamate ionotropic receptor NMDA type subunit 3A (Grin3a)/ 205 bp	NM_138546.2	Forward: GAAGAAAAGCAGCCACGTTC Reverse: GGTTTTGTCCTTCCTCGTCA	55, 20	94.1/0.967
Rattus norvegicus glutamate ionotropic receptor AMPA type subunit 1 (Gria1)/ 114 bp	NM_031608.1	Forward: GGCTCCCTTGACCATAACCT Reverse: ACACCTGGCTTGGACTTCTG	59, 20	100.74/0.994
Rattus norvegicus glutamate ionotropic receptor AMPA type subunit 2 (Gria2)/ 145 bp	NM_017261	Forward: GCATCGCCACACCTAAAGGA Reverse: TTACTTCCCGAGTCCTTGGC	58, 20	97.37/0.994
Rattus norvegicus glutamate ionotropic receptor AMPA type subunit 3 (Gria3)/ 183 bp	NM_001112742.1	Forward: CTCCTGATCCTCCCAATGAA Reverse: TCAGGAAAGCAGCAAGGTTT	55, 15	101.14/0.994
